# An Agent-Based Model of Combination Oncolytic Viral Therapy and Anti-PD-1 Immunotherapy Reveals the Importance of Spatial Location When Treating Glioblastoma

**DOI:** 10.3390/cancers13215314

**Published:** 2021-10-22

**Authors:** Kathleen M. Storey, Trachette L. Jackson

**Affiliations:** 1 Department of Mathematics, Lafayette College, Easton, PA 18042, USA; 2 Department of Mathematics, University of Michigan, Ann Arbor, MI 48109, USA; tjacks@umich.edu

**Keywords:** mathematical modeling, agent-based modeling, oncolytic viral therapy, immune checkpoint inhibitor, combination therapy, glioblastoma

## Abstract

**Simple Summary:**

A combination of oncolytic viral therapy and immunotherapy provides an alternative option to the standard of care for treating the lethal brain tumor glioblastoma (GBM). Although this combination therapy shows promise, there are many unknown questions regarding how the tumor landscape and spatial dosing strategies impact the effectiveness of the treatment. Our study aims to shed light on these questions using a novel spatially explicit computational model of GBM response to treatment. Our results suggest that oncolytic viral dosing in the location of highest tumor cell density leads to substantial tumor size reduction over viral dosing in the center of the tumor. These results can help to inform future clinical trials and more effective treatment strategies for oncolytic viral therapy in GBM patients.

**Abstract:**

Oncolytic viral therapies and immunotherapies are of growing clinical interest due to their selectivity for tumor cells over healthy cells and their immunostimulatory properties. These treatment modalities provide promising alternatives to the standard of care, particularly for cancers with poor prognoses, such as the lethal brain tumor glioblastoma (GBM). However, uncertainty remains regarding optimal dosing strategies, including how the spatial location of viral doses impacts therapeutic efficacy and tumor landscape characteristics that are most conducive to producing an effective immune response. We develop a three-dimensional agent-based model (ABM) of GBM undergoing treatment with a combination of an oncolytic Herpes Simplex Virus and an anti-PD-1 immunotherapy. We use a mechanistic approach to model the interactions between distinct populations of immune cells, incorporating both innate and adaptive immune responses to oncolytic viral therapy and including a mechanism of adaptive immune suppression via the PD-1/PD-L1 checkpoint pathway. We utilize the spatially explicit nature of the ABM to determine optimal viral dosing in both the temporal and spatial contexts. After proposing an adaptive viral dosing strategy that chooses to dose sites at the location of highest tumor cell density, we find that, in most cases, this adaptive strategy produces a more effective treatment outcome than repeatedly dosing in the center of the tumor.

## 1. Introduction

Glioblastoma (GBM) is the most aggressive form of brain tumor, and its poor average survival rate of 1–2 years has remained largely constant for many years, despite significant advancements in other cancer treatment modalities [1]. The standard treatment regimen for GBM consists of surgery, followed by radiotherapy and chemotherapy. Oncolytic viral therapy (OVT) is a treatment involving the administration of a virus that infects and replicates within cancer cells, causing them to lyse. Due to oncolytic viruses’ ability to selectively target tumor cells over healthy cells, OVT has the potential to perform better than the standard GBM treatment protocol when it is combined with an anti-PD-1 immunotherapy, a treatment that targets the protein PD-1, primarily found on T cells. This treatment blocks the immune checkpoint formed when PD-1 binds to PD-L1, which is upregulated on cancer cells. Combining OVT with anti-PD-1 immunotherapy enables a robust immune response to treatment, which aids in targeting tumor cells.

In this work, we develop a spatially explicit model of tumor response to a combination of OVT and immunotherapy, which allows us to study the impact of oncolytic viral dosing locations. This model adds to a growing body of literature featuring mathematical models of OVT. D. Wodarz has made significant contributions to this field, initially developing a model to study the virus-specific and tumor-specific adaptive immune responses to OVT in [2]. Later, he and N. Komarova developed a general framework to study oncolytic viral dynamics in [3,4]. R. Eftimie and colleagues extended this work by focusing on the interactions between an oncolytic virus and T cells, and the resulting multistability and multi-instability [5]. In [6], R. and G. Eftimie investigate the impact of two distinct types of macrophage, M1 and M2, on OVT, and found that polarization toward either type can enhance OVT, in one case through antitumor immune activity, and in the other through elevated cytotoxic activity. A. Friedman and colleagues studied the effect of an immunosuppressive drug, when combined with OVT, in glioma patients, and they concluded that this drug increases the percentage of infected tumor cells in the tumor microenvironment (TME) [7]. We utilize findings from these models comprised of ordinary differential equations to develop a spatially explicit model that provides a more realistic representation of the spatial heterogeneity found in most tumors.

The agent-based model (ABM) has emerged as a premier tool in systems biology and translational oncology, due to its ability to provide a more realistic approximation of interconnected cellular interactions and the complex spatial structure of the TME [8,9]. ABMs can be developed based on biological mechanisms inferred from experiments, with model simulations then used to validate the original datasets, as was carried out using a model of human adipose-derived stromal cell trafficking during acute skeletal muscle ischemia in [10]. In [11], the authors developed a translational ABM of liver fibrosis, combining molecular and histopathological characteristics, which they used to test potential antifibrotic strategies. ABMs have also been used to investigate the immune response to various cancer treatements; in [12], G. Chang et al. study the spatial patterns of PD-L1, a molecule involved in immune checkpoints, which provides a framework to test and compare predictive biomarkers for various cancer treatments. L.G. de Pillis et al. developed a hybrid PDE-cellular automata model of tumor–immune interactions in [13,14], which we used as inspiration for some of the interaction rules in our model. D.R. Berg and colleagues compare the results from oncolytic viral spread in two-dimensional and three-dimensional ABMs in [15], revealing the importance of the spatial structure in OVT efficacy.

In this work, we develop a novel multiscale hybrid ABM and partial differential equations (PDE) model that incorporates tumor cells, innate and adaptive immune cells, and treatment with an oncolytic virus and anti-PD-1 immunotherapy. We developed this three-dimensional model in order to investigate the role of space in oncolytic viral dosing strategies and interactions between the virus and immune and tumor cells. Oncolytic viruses are typically administered in the same location within the tumor, so we are particularly interested in using the model to study alternative dosing strategies and the importance of the dosing location in tumor size reduction.

The outline of this paper is as follows: In Section 2 we describe the development of our multiscale agent-based model and the rules that govern each cell within the model. In Section 3, we present our results regarding the importance of T cells killing vs. tumor-mediated T cell proliferation, and the importance of viral dosing in the locations of the highest cell density. We discuss the implications of our results and describe future directions in Section 4.

## 2. Materials and Methods

In this work, we develop a spatially explicit hybrid cellular automaton (CA) and partial differential equations (PDE) model. We simulate the model in a three-dimensional domain, $\Omega ={\left[0,L\right]}^{3}$. Each site in the lattice can be occupied by at most one tumor cell, one innate immune cell, and one T cell. Note that the average diameter of a GBM cell (line U87) is 12–14 μm [16], the average diameter of a T cell is 7–10 μm, and the average diameter of a macrophages is 21 μm [17]. Due to the similar scale between all three cell types, it is reasonable to model them on the same lattice. We define the three-dimensional neighborhood of each site to be the Moore neighborhood, i.e., the nearest 26 sites.

We model the virus using a reaction-diffusion PDE, which we describe in more detail in Section 2.3. The virus is initially injected into the center of the initial population of susceptible tumor cells, which we initialize as the center of the domain. Thus, we start with nonzero viral concentration in a spherical region at the center of the tumor, with zero concentration everywhere else. We start with 1000 tumor cells and administer a dose of $10^{6}$ pfu, which are scaled analogously from our ODE model in [18]. Each viral dose spans a region a radius of 2 sites, corresponding to 33 total sites in the model.

**Figure 1:**
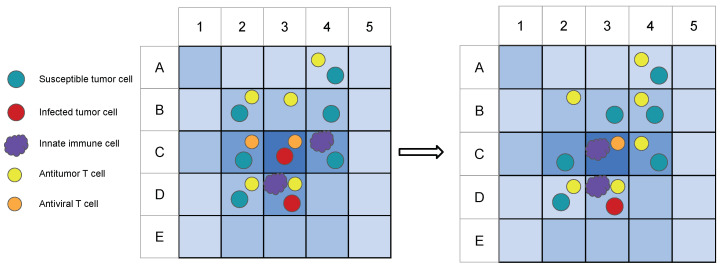
An example of a two-dimensional cross-section of the ABM before (**left**) and after (**right**) a time step τ. The blue shades in the background indicate the viral concentration levels at each site, with darker shades representing higher viral concentrations. In this example, the T cell in A4 replicates and places a daughter T cell in B4, the antitumor T cell in B2 kills the susceptible tumor cell, the T cell in B3 moves to C4, the tumor cell in B4 replicates and places a daughter cell in B3, the T cell in C2 dies naturally, the antiviral T cell in C3 kills the infected cell, the innate immune cell in C4 moves to C3, the viral concentration in D2 decreases due to T cell consumption, and the viral concentration in D3 decreases due to innate immune cell consumption.

To give the reader an idea of the cell actions within the model, Figure 1 displays a schematic example of a two-dimensional cross-sectional portion of the ABM before and after a time step τ. The blue shades indicate viral concentration levels, and the shapes represent tumor and immune cells within the model. The events taking place within this time step include tumor and T cell division, T cell killing of tumor cells, immune cell migration, natural cell death, and immune cell consumption of viral particles.

### 2.1. Tumor Cells

Tumor cells can become infected by the virus in a neighborhood of its location, so we first define N(x,t) to denote the Moore neighborhood of x at time *t*, i.e., the 26 nearest sites in the three-dimensional domain. The probability that susceptible tumor cells at site x can become infected during the time step [t,t+τ) is defined as a function of the viral concentration at that site, V(x,t), as follows:

(1) $$\begin{array}{c} P_{inf}\left(V\left(x,t\right)\right)=\tau \beta T_{s}\left(t\right)V\left(x,t\right), \end{array}$$

where β is the infection rate (pfu${}^{-1}$h${}^{-1}$), used in the ODE model in [18], and $T_{s}\left(t\right)$ denotes the total number of susceptible tumor cells, $T_{s}\left(t\right)=\sum_{x\in \Omega }T_{s}\left(x,t\right)$.

For simplicity, we assume no tumor cell motility, so the tumor cells spread outward exclusively through cell division. Each new tumor cell is assigned an intrinsic cell cycle time, normally distributed with a mean of $\ln\left(2\right)/r_{t}$, where $r_{t}$ is the growth rate of tumor cells (h${}^{-1}$), and standard deviation $\sigma_{cycle}$. At each time step, the cell cycle clock decreases by the time step length with probability $P_{div}\left(t\right)$, defined below. A given cell divides once its cycle clock surpasses 0. Note the mean cell cycle time, $\mu_{cycle}$, is converted from the fitted growth rate $r_{t}=0.0192$ h${}^{-1}$ in [18], and $\sigma_{cycle}=3$ h is an ad hoc estimate. When a susceptible tumor cell divides, it randomly chooses one of its neighboring sites, unoccupied by another tumor cell, on which to place its daughter cell. If there are no unoccupied sites, then it randomly chooses one of its 26 nearest neighbors to push outward to make room for its daughter cell, and then a chain of cells is pushed outward in the same direction. Such cell division assumptions are consistent with those used in the ABM in [19].

In order to enforce the tumor carrying capacity, we define the following probability of reducing each cell division counter at time *t*, as a function of the susceptible and infected tumor populations:

(2) $$\begin{array}{c} P_{div}\left(t\right)=\exp\left(-\gamma_{div}\left(T_{s}\left(t\right)+T_{I}\left(t\right)\right)\right), \end{array}$$

where as above, $T_{s}\left(t\right)$ refers to the total number of susceptible cells, and similarly, $T_{I}\left(t\right)=\sum_{x\in \Omega }I\left(x,t\right)$ denotes the total number of infected cells at time *t*. This assumption is consistent with those made in [13,14,20], and results from these models have shown agreement with experimental data cited in [14,21].

### 2.2. Immune Cells

Innate immune cells arise, i.e., become “activated” to target viral antigens, at a site x which is unoccupied by an innate immune cell, with a probability $P_{inn}$. This probability depends primarily on the viral concentration at each site, as defined below. Additionally, there is a positive feedback loop of recruitment between macrophages and natural killer cells, so the probability also depends on the current population of innate immune cells in neighboring sites of x. We define

(3) $$\begin{array}{c} P_{inn}\left(x,t\right)=1-\exp(-\gamma_{i_{1}}V\left(x,t\right)-\gamma_{i_{2}}Z\left(N\left(x,t\right)\right), \end{array}$$

where, as above, N(x,t) denotes the neighborhood of x at time *t*. We estimate $\gamma_{i_{1}}$ and $\gamma_{i_{2}}$ so that $\gamma_{i_{1}}V\left(x,t\right)$ is generally larger than $\gamma_{i_{2}}Z\left(N\left(x,t\right)\right)$ in the presence of the virus. We use a similar assumption to the models used in [13,14], adapted to account for the concentration of virus at each site.

Adaptive immune cells, i.e., T cells, are recruited to the tumor microenvironment (TME) by the innate immune cells. At each site occupied by an innate immune cell and currently unoccupied by a T cell, there is a probability of $\tau a_{AZ}$ that a T cell will be recruited to this site, during the time interval [t,t+τ). The type of T cell, i.e., antiviral vs. antitumor, recruited to this site is determined globally. This status depends on the proportion of total susceptible cells, $T_{s}\left(t\right)$, to infected tumor cells, $T_{I}\left(t\right)$, in the domain. If $T_{s}\left(t\right)+T_{I}\left(t\right)>0$, then the probability that a new T cell will be antiviral is

(4) $$\begin{array}{c} P_{A_{V}}\left(t\right)=1-\exp\left(-\gamma_{A_{V}}\frac{T_{I}\left(t\right)}{T_{I}\left(t\right)+T_{s}\left(t\right)}\right), \end{array}$$

and $1-P_{A_{V}}\left(t\right)$ is the probability that the T cell will be antitumor. Note we are assuming that the tumor vasculature exists throughout the TME, so tumor infiltration by immune cells occurs via the vasculature, as described in [22]. This allows immune cells to emerge at sites within the tumor, rather than starting on the boundary of the tumor.

We also incorporate innate and adaptive immune motility within the model. Innate and adaptive immune cells move at distinct rates and randomly choose a neighboring site that is unoccupied by an immune cell of its type. The immune cells move preferentially toward the cells that they target, modeling chemotaxis. Hence, innate immune cells and antiviral T cells are $m_{Z}$ and $m_{A_{V}}$ times more likely, respectively, to move toward sites occupied by infected tumor cells than to other sites, and antitumor T cells are $m_{A_{T}}$ times more likely to move toward sites occupied by tumor cells than to other sites. Note that $m_{Z}$, $m_{A_{V}}$, $m_{A_{T}}$ are all larger than 1. Innate immune cells move to an empty neighboring site, i.e., one that is not currently occupied by an innate immune cell, at rate $r_{mov_{Z}}$. Thus, the probability that such an innate immune cell moves to a neighboring site in the time interval [t,t+τ) is $\tau r_{mov_{Z}}.$ Subsequently the innate immune cell chooses an empty site to migrate to, and it is $m_{Z}$ times more likely to move to a site occupied by an infected cell than any other site. Similarly, the antitumor and antiviral T cells move at rates $r_{mov_{AT}}$ and $r_{mov_{AV}}$, respectively.

To simulate T cell proliferation, if an antitumor or antiviral T cell and tumor cell are located at the same site, the T cell proliferates and adds a daughter cell to a randomly chosen neighboring site during time interval [t,t+τ) with probability $\tau a_{AT}$ or $\tau a_{AV}$, respectively. Here, $a_{AT}$ and $a_{AV}$ are the rates of the tumor cell- or infected cell-mediated proliferation of T cells, respectively, from the ODE model in [18], which we have converted to probabilities in this stochastic ABM. Similarly, innate immune cells at the same site as an infected tumor cell proliferate at rate $a_{Z}$, so the probability of such a proliferation event at the site of an innate immune cell and infected tumor cell in [t,t+τ) is $\tau a_{Z}T_{I}\left(t\right)$.

There are two mechanisms of immune cell death: one is driven by a natural death rate, causing cells to die after a certain amount of time, and the second is driven by immune activity, causing cells to die after a certain number of immune “events”, modeling the exhaustion of the cells. For simplicity, we assume that the immune events are restricted to the killing of tumor cells, since the killing of viral particles is difficult to measure as individual events.

If an adaptive immune cell and tumor cell simultaneously occupy the same site, i.e., an antiviral T cell with an infected tumor cell or an antitumor T cell with any tumor cell, then the tumor cell dies at rate $k_{IA}$ or $k_{TA}$ cell${}^{-1}$h${}^{-1}$, respectively. Hence, the probability of such an event in [t,t+τ) is $\tau k_{TA}$ or $\tau k_{IA}$. Similarly, the probability that an innate immune cell kills an infected tumor cell at the same site in [t,t+τ) is $\tau k_{I}$, where $k_{TA},k_{IA},k_{I}$ are all rates from the ODE model in [18].

**Figure 2:**
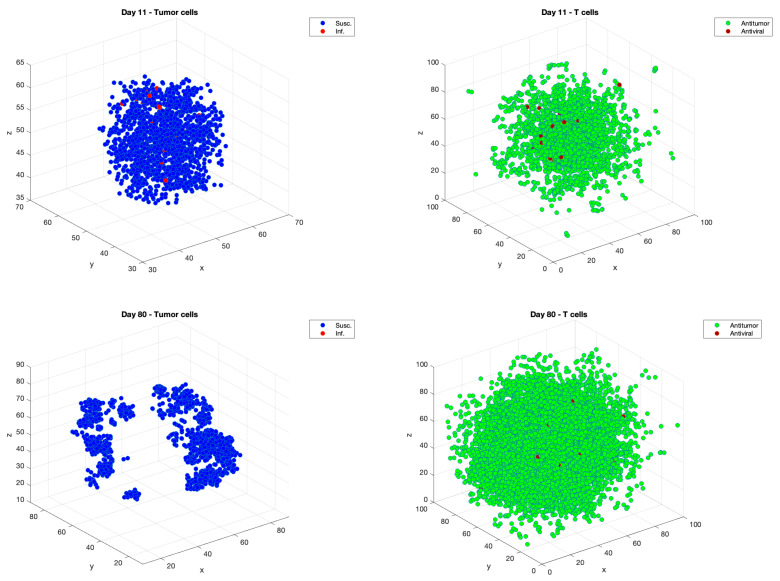
Examples of 3-D simulations plots of tumor cells and T cells on Day 11, immediately after Dose 2, in the top row, and on Day 80, at the end of the simulation, on the second row. On Day 11, there are $1.51\times 10^{3}$ tumor cells and $1.44\times 10^{4}$ T cells. On Day 80, there are $1.40\times 10^{3}$ tumor cells and $5.83\times 10^{4}$ T cells.

Figure 2 illustrates the three-dimensional locations of infected and susceptible tumor cells, on the left, and antitumor and antiviral T cells, on the right, on days 11, immediately after Dose 2 is administered, and on Day 80, at the end of a sample simulation.

We also incorporate PD-1/PD-L1 checkpoints in the ABM, similarly to our ODE model in [18]. The PD-1/PD-L1 checkpoint implementation is described in more detail in Appendix A.1. The immune checkpoints can be blocked by anti-PD-1 immunotherapy when it has been administered. We incorporate treatment with anti-PD-1 using a global approach, i.e., we calculate the blocking rate of PD-1 using the total concentration of PD-1 in the tumor microenvironment at time *t*. Further detail describing this implementation can be found in Appendix A.2.

### 2.3. Viral Diffusion

The viral concentration will be modeled using a reaction-diffusion equation, as follows:

(5) $$\begin{array}{c} \frac{\partial V(x,t)}{\partial t}=D_{V}\Delta^{2}V\left(x,t\right)-\omega V\left(x,t\right)+b_{T}\delta_{T}T_{I}\left(x,t\right)-k_{VZ}Z\left(x,t\right)V\left(x,t\right)-k_{VA}Y_{V}\left(x,t\right)V\left(x,t\right), \end{array}$$

where $D_{V}$ is the diffusion coefficient for the virus, and $k_{VZ}Z\left(x,t\right)$ and $k_{VA}Y_{V}\left(x,t\right)$ represent viral death due to consumption by innate immune cells and killing by adaptive immune cells, respectively, at rates $k_{VZ}$ and $k_{VA}$.

Additionally, $\alpha_{I}\beta T_{s}V$ represents viral infection of susceptible tumor cells at rate $\beta_{T}$. The term $b_{T}\delta_{T}T_{I}\left(x,t\right)$ represents the viral particles that are released when an infected cell bursts at rate $\delta_{T}$, with burst size $b_{T}$. We assume that the virus cannot leave or enter from the boundary, so the boundary conditions are:

V(0,y,t)=V(L,y,t)=V(x,0,t)=V(x,L,t)=0.

We discretize Equation (5) using central differences for spatial derivatives and forward differences for time derivatives (FTCS).

## 3. Results

### 3.1. Antitumor T Cell Killing

**Figure 3:**
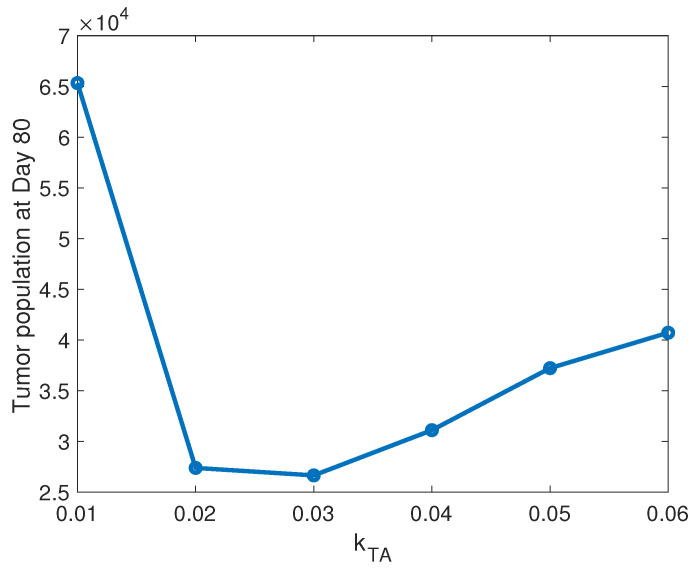
Antitumor T cell killing rate. This figure depicts the mean tumor population at Day 80, as a function the tumor cell killing rate by antitumor T cells, $k_{TA}$. Means are calculated from 10 simulations performed for $k_{TA}=\left[0.01,0.02,0.03,0.04,0.05,0.06\right]$, with all other parameters set at their baseline levels, including $a_{AT}=0.025$.

We utilize the model to gain a better understanding of the role of T cells in response to the combination therapy, by comparing the relative importance of hte T cell-mediated tumor killing rate with tumor antigenicity. First, we investigate the impact of the tumor cell killing rate, $k_{TA}$, by antitumor T cells on the tumor response to treatment. We vary the parameter $k_{TA}$ in the range [0.01,0.06], around the baseline value of 1/24≈0.42 cell${}^{-1}$h${}^{-1}$, estimated from [23], leaving all other parameters set at their baseline rates. Figure 3 shows the susceptible tumor population at Day 80, the end of each simulation, as a function of $k_{TA}$. Interestingly, we observe a nonmonotonic relationship between tumor size and T cell killing rate. As one would expect, for the lowest killing rate in the range under consideration, $k_{TA}=0.01$ cell${}^{-1}$h${}^{-1}$, the T cells are not sufficiently cytotoxic to respond effectively to the treatment, leading to a significantly larger tumor after 80 days than we see with larger $k_{TA}$ values. The tumor decreases to similar levels for $k_{TA}=0.02$ and 0.03, with a slightly smaller population for $k_{TA}=0.03$. However, as $k_{TA}$ increases further, the tumor population increases as well, suggesting that increasing the T cell cytotoxicity beyond a certain threshold will not improve the efficacy of the treatment. We hypothesize that in this high $k_{TA}$ range, the T cells kill cancer cells too quickly early in the treatment process, which ultimately leads to a smaller number of T cells to mount an attack on the tumor. This investigation suggests an optimal value of $k_{TA}=0.03$, at which the tumor population initially increases enough to activate a sufficient level of antitumor T cells, which in turn keep the tumor relatively controlled.

### 3.2. Tumor-Mediated T Cell Proliferation

**Figure 4:**
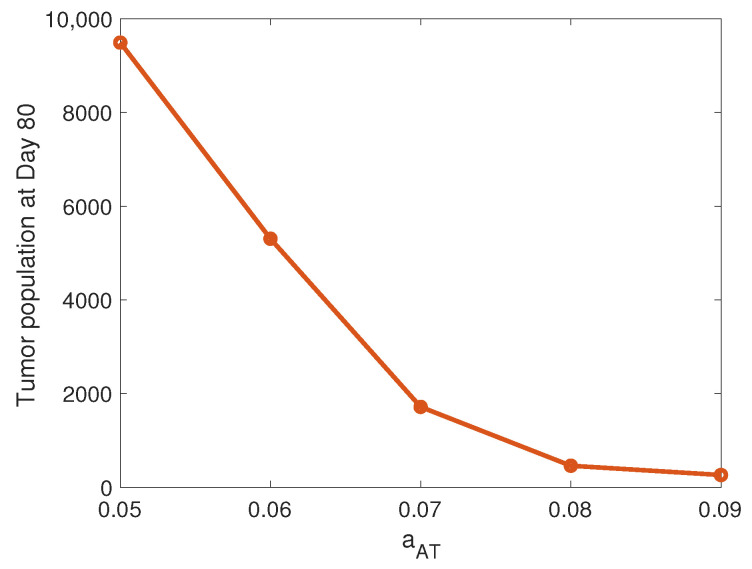
Tumor-mediated T cell proliferation. This figure depicts the mean tumor population at Day 80, as a function the tumor-mediated T cell proliferation rate, $a_{AT}$. Means are calculated from 10 simulations performed for $a_{AT}=\left[0.05,0.06,0.07,0.08,0.09\right]$, with $k_{TA}=0.03$ and all other parameters set at their baseline levels.

We compare the relative impact of the T cell killing rate from the previous section with the level of tumor antigenicity. In our model, this antigenicity level is dictated by the tumor-mediated proliferation rate of T cells, $a_{AT}$, which we found to be a significant adaptive immune-related parameter in our ODE model parameter sensitivity analysis in [18]. Figure 4 displays the susceptible tumor population at the end of the simulation as a function of $a_{AT}$. We observe a monotonic relationship between the final tumor size and $a_{AT}$, with the tumor decreasing as the level of tumor antigenicity increases. Note that high levels $a_{AT}$ can lead to a small, well-controlled tumor 80 days after the start of treatment, around 450 cells for $a_{AT}=0.08$ and 260 cells for $a_{AT}=0.09$ after starting the simulations with about 1000 cells. We did not observe this significant reduction in tumor size at the optimal value for $k_{TA}$. This suggests that the treatment is more effective when there is a critical mass of T cells to attack the tumor than when there is a smaller number of highly cytotoxic T cells. Building this critical mass of T cells seems to be particularly important during the early stages of the simulation, when the tumor increases rapidly until the T cells are able to mount a sufficient attack on the tumor cells.

**Figure 5:**
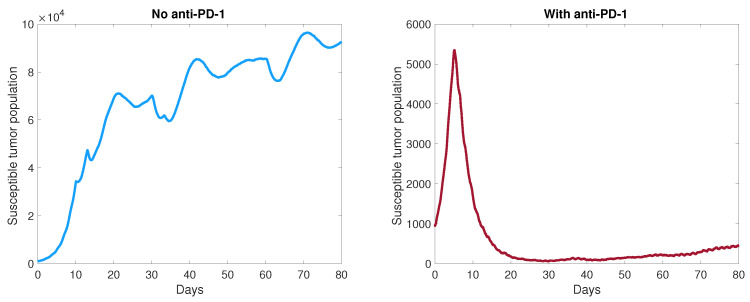
Comparison with and without anti-PD-1. Simulations using the same parameter values, with $a_{AT}=0.08$.

In Figure 5, the graph on the right shows the tumor control achieved from the combination of six oncolytic viral doses administered in the center of the domain and anti-PD-1 immunotherapy, using a representative simulation with $a_{AT}=0.08$. The graph on the left shows the susceptible tumor population when oncolytic viral therapy is administered without anti-PD-1. In this case, the tumor size trends upward with short sporadic periods of decline, until it starts to level off toward the end of the simulation. Overall, we see no tumor control without the addition of anti-PD-1, and similar behavior is observed for other parameter sets. Thus, it is imperative to administer an anti-PD-1 immunotherapy in combination with OVT, so that the immune response stimulated by OVT can be sufficiently effective.

### 3.3. Viral Dose Timing

**Figure 6:**
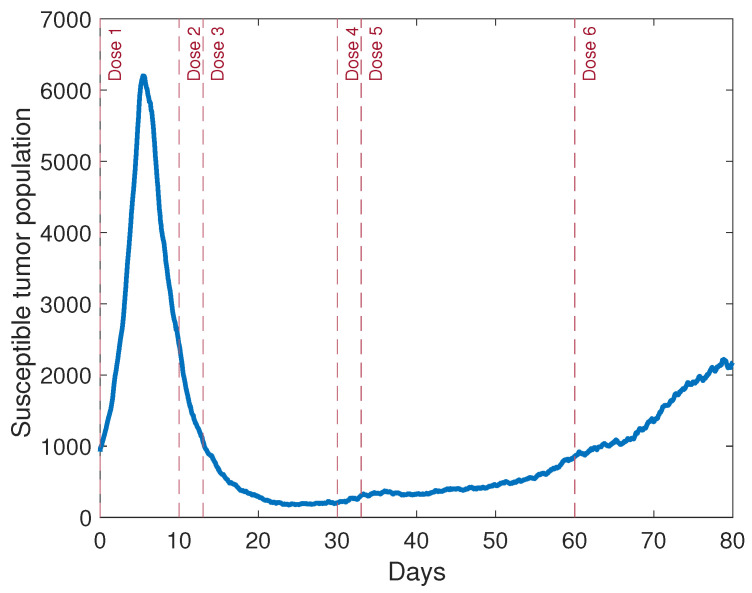
A simulation of the ABM with the chosen viral dosing schedule obtained from sequentially testing each dose. In this simulation, $a_{AT}=0.07$, and all other parameters are set at their baseline levels.

Thus far, there has been little focus in the literature on the timing of oncolytic viral doses, but timing can be crucial to treatment efficacy, especially when a multifaceted immune response plays a significant role, as it does in the case of OVT. Thus, we used our model to sequentially investigate the optimal timing of six viral doses. We fixed the initial viral dose at the start of the simulation, and then we varied the timing of viral dose 2, at Day 4, the approximate time of the population peak when a single viral dose is administered, at Day 10, Day 15, and Day 20. After comparing the means from 10 simulations for each option, we found that dosing at Day 10, about six days after the tumor population begins declining, produces the smallest tumor at the end of the simulation. Thus, we fixed Dose 2 at Day 10, and subsequently tested Dose 3 at Days 13, 20, 30, and 40, following the same procedure that we used to determine the timing of Dose 2. Our results suggested that it was optimal to administer Dose 3 shortly after Dose 2, on Day 13. By using a similar process for Doses 4–6, we determined that it was optimal to administer Dose 4 at Day 30, Dose 5 at Day 33, and Dose 6 at Day 60. Thus, our results suggest that the combination therapy may be more effective when pairs of viral doses are given close together, followed by a longer break before the next pair of doses. The timing of the doses is indicated in Figure 6, which displays a simulation of the tumor, with tumor antigenicity level $a_{AT}=0.07$ and all other parameters set at their baseline levels.

### 3.4. Density-Based Adaptive Viral Dosing

The location of oncolytic viral doses has also not been well-explored, and can be consequential for developing an effective treatment regimen. Our spatially explicit ABM proves particularly useful for investigating the effect of the viral dosing locations on the treatment response. We model the direct intratumoral delivery of the virus, which generally controls the viral concentration more effectively in a specific location than intravenous delivery [24]. Typically, the intratumoral dose is administered roughly in the center of the tumor region, which we simulated in the model by administering each viral dose in the center of the three-dimensional domain. We then compared the center dosing results with an adaptive dosing strategy, in which viral doses were administered at the locations of maximum tumor cell density at the time of each dose. We implemented this in the model by calculating the cell density in a three-dimensional neighborhood with a two-site radius around each site in the domain. We chose the site with maximum tumor cell density to administer the viral dose; if there is more than one site with maximal cell density, then we split the viral dose evenly between these sites.

**Figure 7:**
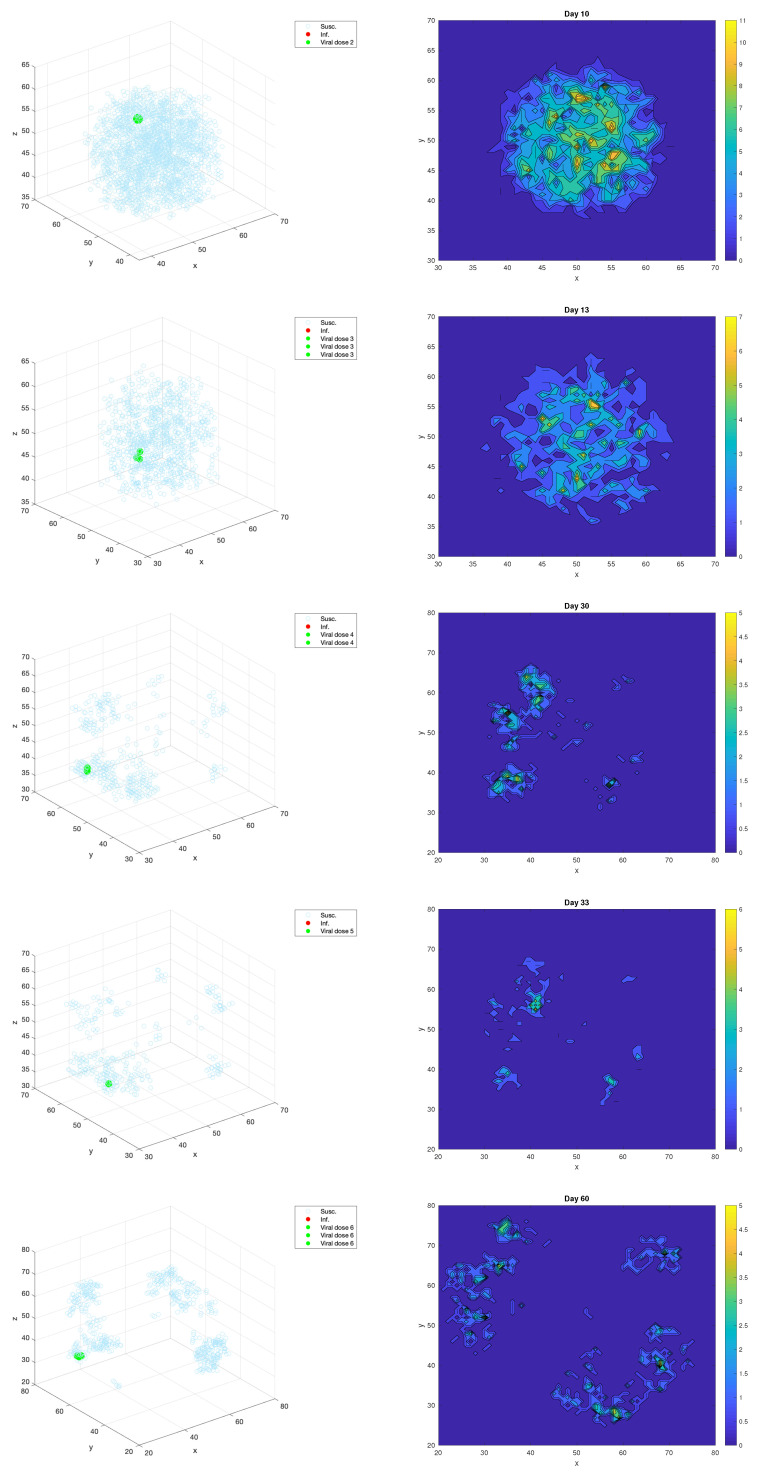
The chosen viral dosing locations for Doses 2–6 in the realization of the ABM. The locations in the 3-D domain are shown on the left, while the two-dimensional tumor cell density is shown on the right, obtained from projecting the *z* dimension onto the xy-plane.

Figure 7 shows an example of the adaptive dosing procedure for the realization of the model with tumor antigenicity level, $a_{AT}=0.07$. Each row shows the chosen location(s) for Doses 2–6, with the three-dimensional plot on the left, showing the susceptible tumor cells in light blue and the dosing locations highlighted in green. In order to more easily visualize the dense regions, the plots on the right of the figure display the two-dimensional tumor cell density resulting from the projection of the *z*-dimension onto the xy-plane. For Dose 2 on Day 10, the chosen dosing location in the xy-plane is (52,57), which corresponds to a high density level in the 2-D projection. There are three chosen dosing locations for Dose 3, which all have a neighborhood with the maximum tumor cell density level, and these are all neighboring sites: (46,51),(46,52),(47,52) in the xy-plane. Similarly, two neighboring sites are chosen for Dose 4, at (40,63) and (41,64) in the xy-plane. Dose 5 is chosen to be administered at site (41,56), and Dose 6 is split between the three neighboring sites at (34,73),(34,74), and (35,74). We note that even when multiple sites have neighborhoods with the same maximum tumor cell density, they are always neighboring sites in this example, and they are nearly always neighboring sites in the other realizations that we have simulated. Thus, in a clinical setting, it is reasonable to choose a single location of estimated highest tumor cell density using tumor imaging technology.

**Figure 8:**
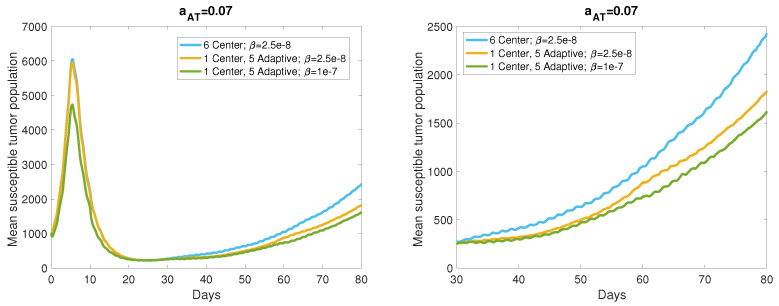
Means generated from 40 realizations for each parameter set, with antigenicity level $a_{AT}=0.07$. The blue curve is generated from simulations with six center viral doses and viral infection rate, $\beta =2.5\times 10^{-8}$ pfu${}^{-1}$h${}^{-1}$, the yellow curve is generated from simulations with one center dose and five adaptive doses and $\beta =2.5\times 10^{-8}$, and the green curve is generated from simulations with one center dose and five adaptive doses and $\beta =1\times 10^{-7}$.The graph on the right is a zoomed in version of the full trajectories on the left.

We compared the adaptive viral dosing strategy to center dosing in the range of antigenicity levels leading to a responsive or stable tumor, characterized by at least 50% tumor reduction from its maximum size, by the simulation end time, 80 days after the start of treatment. These response levels are reached with $a_{AT}=0.07,0.08$, so we simulated 40 realizations of the ABM for each of these antigenicity levels to account for the stochasticity within the model. Figure 8 displays the mean tumor size resulting from these realizations, with the center dosing mean shown in blue and the adaptive dosing mean shown in yellow. The graph on the right is a zoomed in version of the left graph to highlight the difference in tumor size at the end of the simulation. We also investigated the benefit conferred by increasing the viral infection rate to $\beta =1\times 10^{-7}$ pfu${}^{-1}$h${}^{-1}$, with the mean tumor size shown by the green curve in this figure. The average tumor size 80 days after the start of treatment with center dosing is $2.425\times 10^{3}$ cells, and adaptive viral dosing yields a 24.5% reduction in this average tumor size. Increasing the viral infection rate to $1\times 10^{-7}$ pfu${}^{-1}$h${}^{-1}$ yields an additional 8.6% reduction in average tumor size over the center dosing case. Hence, we observe a sizable improvement in treatment response due to adaptive dosing in locations of high tumor cell density, with a marginal improvement when the virus is highly infectious.

**Figure 9:**
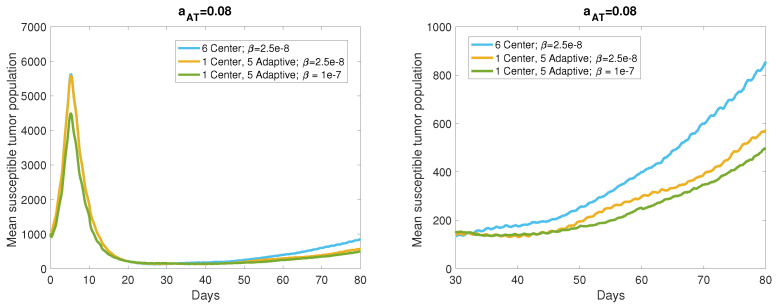
Means generated from 40 realizations for each parameter set, with antigenicity level $a_{AT}=0.08$. The blue curve is generated from simulations with six center viral doses and viral infection rate, $\beta =2.5\times 10^{-8}$ pfu${}^{-1}$h${}^{-1}$, the yellow curve is generated from simulations with one center dose and five adaptive doses and $\beta =2.5\times 10^{-8}$, and the green curve is generated from simulations with one center dose and five adaptive doses and $\beta =1\times 10^{-7}$. The graph on the right is a zoomed in version of the full trajectories on the left.

Similarly, Figure 9 shows the tumor population means for the same cases, with a higher level of tumor antigenicity, $a_{AT}=0.08$ cell${}^{-1}$h${}^{-1}$. In this case, we see even more improvement resulting from the adaptive dosing strategy. The mean tumor size 80 days after the start of treatment with center dosing is 855.3 cells, and the adaptive dosing strategy reduces this mean by 33.4%, which is comparable to the reduction resulting from both adaptive dosing and increased viral infectivity in the $a_{AT}=0.07$ case. Increasing the viral infection rate when $a_{AT}=0.08$ provides an additional 8.8% reduction in tumor size over the center dosing mean. These results suggest that choosing viral dosing locations in the regions of highest cell density produces significant improvement over repeatedly dosing in the same location, and we observe from Figure 8 and Figure 9 that this improvement appears to increase over time. Note that for $a_{AT}\geq 0.09$, both center and adaptive viral dosing produce near tumor clearance, so adaptive dosing does not provide a significant improvement when center dosing yields such a strong treatment response.

**Figure 10:**
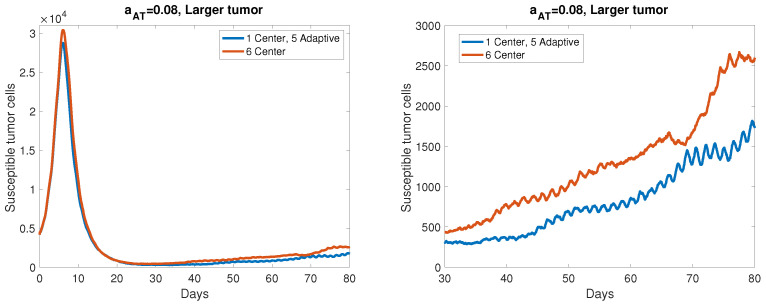
A comparison between center dosing and adaptive dosing in a larger tumor, with $a_{AT}=0.08$. The single simulation starts with a tumor radius of 10 tumor cells, or 4169 total cells, about 4 times the initial susceptible tumor population that we use in our baseline setup.

In order to make model simulations computationally tractable, we were required to scale down the initial tumor population to start around 1000 cells, which is two orders of magnitude smaller the initial tumor population we used in the ODE model in [18]. Similarly we scaled the standard viral dose used in the ODE model by $10^{-2}$ to obtain a proportional viral dose to use in the ABM. To gauge whether we can expect comparable results in a larger tumor, we tested the $a_{AT}=0.08$ case in a tumor starting with 5000 cells, so about 5 times our typical simulation size. In a single simulation, we found the ratio of tumor sizes between center dosing and adaptive dosing to be 0.665, or a nearly identical tumor size reduction of 33.5% with adaptive dosing to the average reduction we observed with the smaller tumors. The results of this simulation are shown in Figure 10, with the graph on the right showing a zoomed in version of the full trajectory on the left. Although this tumor is still significantly smaller than a human tumor in vivo, we are encouraged by the fact that the percent size reduction scales up nearly identically from our typical simulation size in this larger example.

**Figure 11:**
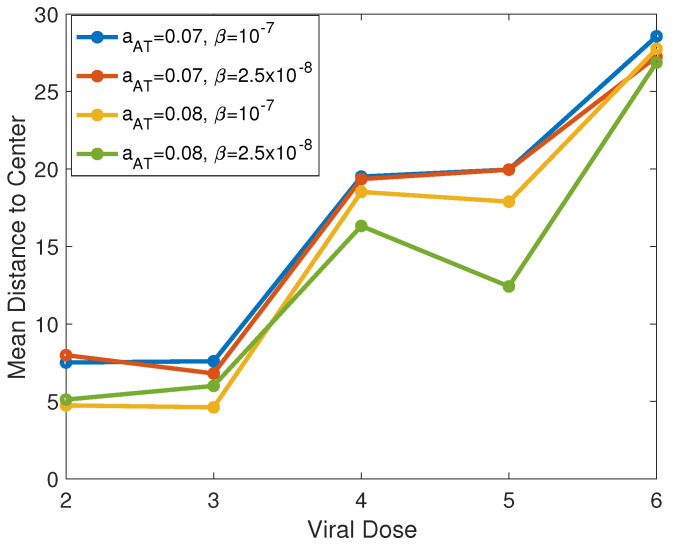
Mean adaptive viral dosing distance to the center of the domain. The means are shown from 40 realizations of each parameter set for adaptive viral doses 2–6.

We also investigate the chosen locations in the adaptive dosing procedure. In order to compare the dosing locations for each dose number, we plot the mean adaptive viral dosing distances from the center of the domain for Doses 2–6, generated from 40 realizations of each parameter set, in Figure 11. We plotted these means for $a_{AT}=0.07,0.08$ and $\beta =2.5\times 10^{-8},1\times 10^{-7}$, and we observed that, in almost all cases, the dosing locations move farther away from the domain center as the dose number increases. Due to the growing difference between the adaptive and center dosing strategies as the dose number increases, this suggests that adaptive viral dosing may confer a more significant benefit in later doses, when the high-density regions have spread farther from the initial tumor center.

## 4. Discussion

Over the past several decades, efforts to develop new therapies for GBM tumors have intensified, yet none have significantly impacted patient mortality [25]. OVs, equipped with specific oncolytic properties, have the potential to confer therapeutic benefits to GBM patients due to their specificity, potency, tolerability, and potential to be combined to with multiple immunotherapies [26,27]. Questions remain surrounding OVT dosing strategies and concerning the precise role of the immune system in response to OVT, in a quest to increase the efficacy of OVT. In order to address these questions, we developed an agent-based model that allows us to consider immune interactions and to investigate the impact of the spatial location of viral dosing.

The agent-based approach allowed us to extend our mechanistic ODE model in [18] to consider more complex spatial interactions. We previously developed the ODE model to study the interactions between GBM cancer cells and innate and adaptive immune cells in response to OVT and anti-PD-1 therapy. A limitation of that mechanistic model was that it could not capture spatial heterogeneity inherent in GBM tumors, including the spatial distribution of various cell types and the diffusion of the virus and anti-PD-1 drug. This motivated us to develop a spatially explicit ABM of tumor response to a combination of oncolytic viral therapy and anti-PD-1 immunotherapy. ABMs are important tools in translational systems biology, due to their ability to incorporate spatial structure and stochasticity on multiple spatial and time scales, and they have become much more prevalent in recent years [9].

Both the ABM and our previous ODE model suggest that anti-PD-1 immunotherapy is necessary to allow the OVT to work effectively, with the body’s T cells serving as the primary antitumor weapon. When OVT is not combined with anti-PD-1, the PD-1/PD-L1 complex prevents many T cells from targeting the tumor cells, resulting in an insufficient immune response. The ABM also confirms our finding using the ODE model, that the tumor antigenicity level is more consequential for OVT treatment response than the T cell killing rate of tumor cells. Thus, if the treatment does not yield a reduction in tumor size shortly after the start of treatment, it may be advantageous to administer an IL-2 immunotherapy in combination with the OVT and anti-PD-1, in order to increase the T cell proliferation rate. Modeling the combination of these three treatment options will be the subject of future work.

An important benefit of the ABM approach is that it allows us to explore spatial strategies for oncolytic viral dosing, which is not possible with mechanistic ODE models. We compare an adaptive dosing strategy that chooses the sites with the highest tumor cell density with a fixed dosing strategy that administers each dose in the same location throughout the treatment period. Given a sufficiently antigenic tumor, the density-based adaptive dosing strategy is more effective than the fixed dosing strategy, yielding a smaller post-treatment tumor. When using the adaptive strategy, each successive viral dosing location is chosen to be farther from the center, as the tumor spreads outward. Hence, the utility of the adaptive dosing strategy is more pronounced for doses later in the treatment schedule. We find that there is a more significant benefit conferred by this adaptive dosing strategy than from using a highly infectious virus. We note that we explored optimal dose timing before our investigation of optimal dose location, so it is possible that the adaptive dosing strategy could be improved even further with the reconsideration of optimal dose timing. In a clinical setting, the dosing locations can be determined using tumor imaging, e.g., computed tomography (CT) scans, which display relative tumor density. Such scans can be used to estimate the location of maximum tumor cell density before each viral dose. Thus, our results suggest that if clinicians have the ability to collect CT scans before each viral dose, then this practice will prove valuable, particularly for doses later in the treatment schedule.

Our ABM allowed us to more accurately approximate tumor growth and local interactions between viral particles, immune cells, and tumor cells. We observed similar overarching trends between the ODE model in [18] and the ABM, particularly with respect to the tumor–immune interactions. Additionally, the spatial features of the ABM allowed us to consider more complex viral dosing strategies. A limitation of the model is that although we did incorporate T cell exhaustion due to tumor cell killing, we did not include a limit on the number of times T cells can proliferate. While we included the PD-1/PD-L1 checkpoint within the model, we did not incorporate other checkpoints like CTLA-4, CD28, and TIM3, which can also impact T cell proliferation. This may result in the model overestimation of the T cell population within the TME. Our model can be easily adapted to explore the impact of additional checkpoints, so in future work, we would like to incorporate these checkpoints and to investigate the effect of a proliferation limit for T cells on the response to this combination therapy. We hope to use this work as motivation to design experiments that test adaptive immune cell killing and proliferation rates and exhaustion-related limits on these processes. This will help to provide further clarity about the feasible ranges for these parameters in the model.

Additionally, the model developed here is parameterized using data from mouse models, so it cannot be directly translated to human patients. We would like to validate our computational results by designing future murine experiments that compare the adaptive and fixed viral dosing strategies, for example, by administering the same combination therapy to each mouse, with the oncolytic virus administered in the tumor center in half of the mice, and the virus administered adaptively in the site of estimated maximum tumor cell density in the other half. The proportional difference between tumor sizes after 80 days can then be compared to our ABM results. We hope that the results will motivate a subsequent clinical trial in GBM patients. In addition, we plan to model the administration of a new type of oncolytic virus that is engineered to express a PD-L1 inhibitor, which provides an opportunity to combine OVT and immunotherapy in a single treatment [28]. The ABM framework is flexible enough to investigate novel therapies such as this one, allowing us to adapt our current ABM for this future work.

## 5. Conclusions

In this study, we developed an ABM to model the response of GBM to a combination of oncolytic viral therapy and anti-PD-1 immunotherapy. Our model simulations suggest that the tumor antigenicity level, which impacts T cell proliferation, is more consequential for treatment efficacy than the T cell killing rate. In addition, we determine an optimal viral dosing schedule and consider an alternative spatial dosing strategy. We show that an adaptive viral dosing strategy that chooses to dose in the locations of highest tumor cell density is substantially more effective than administering each dose in the same location in the center of the tumor. These results suggest that collecting CT scans during treatment for GBM patients can help to inform the location of oncolytic viral dosing, thereby improving the effectiveness of this treatment. 

## Figures and Tables

**Figure A1:**
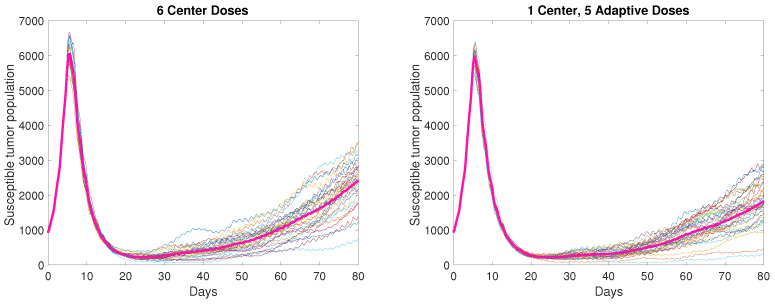
All simulations for $a_{AT}=0.07$, with the thicker pink curves denoting the mean of the trajectories. The left figure shows the simulations with 6 center viral doses administered, and the right figure shows the simulations with 1 center and 5 adaptive doses administered.

**Figure A2:**
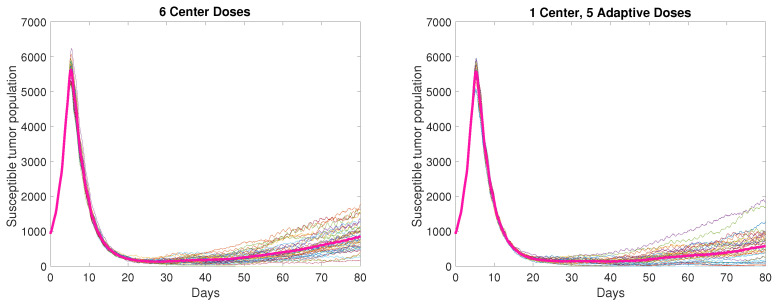
All simulations for $a_{AT}=0.08$, with the thicker pink curves denoting the mean of the trajectories. The left figure shows the simulations with 6 center viral doses administered, and the right figure shows the simulations with 1 center and 5 adaptive doses administered.

## Data Availability

The authors confirm that the data supporting the findings of this study are available within the article. MATLAB code for model generation and simulation can be found at https://github.com/kstorey90/ABM_OVT_antiPD1 (accessed on 10 October 2021).

## Appendix A

For computational feasibility, we initialized the tumor population with about 1000 cells, corresponding to a radius of six sites at the center of the lattice domain. HSV particles have diameters ranging from 155 to 240 nm [29], so we approximate the diameter as 200 nm, which converts to $2\times 10^{-5}$ cm. Using our baseline estimate of l=0.0014 cm for site length, the initial viral dose spans a radius of two sites, corresponding to 33 total sites.

### Appendix A.1. PD-1/PD-L1 Checkpoints

To incorporate PD-1/PD-L1 checkpoints within the model, we multiply the probabilities of the innate immune-mediated activation of adaptive immune cells and of tumor cell-mediated T cell proliferation by a factor F(P,L). This factor represents the suppression of T cell activation and proliferation via the PD-1/PD-L1 checkpoint, where P,L denote the molar concentrations of PD-1 and PD-L1, respectively, expressed by cells within the model. The molar concentrations (in μmol/cm${}^{2}$) are obtained by first calculating the PD-1 expression on all T cells and the PD-L1 expression on all T cells, tumor cells, and innate immune cells, as outlined below. As *P* and *L* increase, so does the number of PD-1/PD-L1 complexes within the tumor region. This increase corresponds to a smaller F(P,L) value, modeling the inhibition of T cell activity.

In [18], we calculate estimates for the parameters used in the factor F(P,L). We estimate the concentration of PD-1 and PD-L1 per T cell to be $\rho_{P}=1.259\times 10^{-11}\;\mu M$ and $\rho_{L}=2.510\times 10^{-11}\;\mu M$, respectively.

We also calculate the parameter $K_{YQ}=\frac{1}{2}\bar{P}\bar{L}$, where $\bar{P}$, $\bar{L}$ denote the carrying capacity for PD-1 and PD-L1, respectively, to be

$$\begin{array}{cc} K_{YQ} & =1.365\times 10^{-12}\;g^{2}/L^{2}\frac{1}{3.165\times 10^{4}\;g/\mathrm{mol}}\cdot \frac{1}{3.328\times 10^{4}\;g/\mathrm{mol}}\cdot 10^{12}\frac{\mu {\mathrm{mol}}^{2}}{{\mathrm{mol}}^{2}}\\ & =1.296\times 10^{-9}\mu M^{2}. \end{array}$$

### Appendix A.2. Anti-PD-1

In the model, we track the concentration of anti-PD-1 in the tumor microenvironment, using μmol/cm${}^{3}$.

For the decay rate of anti-PD-1, we use $\delta_{A}$ in the range 0.0019–0.01 h${}^{-1}$, from [30,31]. Just as in [18], we estimate the anti-PD-1 blocking rate of PD-1, $\mu_{PA}$, by assuming that 10% of the anti-PD-1 drug is used in blocking PD-1 and that 90% degrades naturally. Thus,

$$\frac{\mu_{PA}PA}{0.1}=\frac{\delta_{A}A}{0.9},$$

so we assume that for a steady state $\bar{P}$ and $\delta_{A}=0.0019$,

$$\mu_{PA}=\delta_{A}/\left(9\bar{P}\right)=\frac{0.0019\;{\mathrm{hr}}^{-1}}{9*2.36\times 10^{-5}\mu M}=8.945\;L/\mu \mathrm{mol}/h.$$

In [18], we derived the following conversion from the dosage, *D*, of nivolumab, in mg/kg, to plasma concentration $C_{max}$, in μM = μmol/cm${}^{3}$:

$$C_{max}\left(D\right)=0.139D+0.064.$$

A typical nivolumab regimen consists of a single intravenous dose of 3 mg/kg nivolumab, administered for one hour, once every two weeks. We incorporate this treatment regimen within our model, by adding nivolumab every two weeks, at concentration $C_{max}$(3 mg/kg) = 0.481 μmol/cm${}^{3}$.

### Appendix A.3. Simulation Results

The trajectories are shown for all 40 realizations of the model with $a_{AT}=0.07$ and $a_{AT}=0.08$ in Figure A1 and Figure A2, respectively.

**Table A1:** Baseline parameters in ABM.

	Parameter	Description	Value	Units	Source
1	*l*	Cell size	0.0014	cm	[16]
2	$\mu_{cycle}\left(\sigma_{cycle}\right)$	Mean (st. dev.) cell cycle time	36.1 (3)	Hours	Estim.
3	$D_{V}$	Viral diffusion coefficient	$3.6\times 10^{-4}$	cm${}^{2}$/h	[7]
4	ω	Viral clearance rate	0.025	h${}^{-1}$	[7]
5	$\delta_{T}$	Death rate of infected tumor cells	$\frac{1}{18}$	h${}^{-1}$	[7]
6	$b_{T}$	Burst size of infected cells	50	pfu/cell	[7]
7	$\alpha_{I}$	Virus absorbed by tumor cell during infection	5	pfu/cell	Estim.
8	$\gamma_{div}$	Parameter used in, $P_{div}$, the probability of reducing cell division counter	$1\times 10^{-5}$	1/cell	Estim.
9	β	Viral infection rate	$2.5\times 10^{-8}$	pfu${}^{-1}$h${}^{-1}$	[32], Est.
10	$\gamma_{i_{1}}$	Parameter governing viral-mediated activation of innate immune cells in $P_{inn}$	$5\times 10^{-5}$	1/pfu	Estim.
11	$\gamma_{i_{2}}$	Parameter governing innate immune cell positive feedback in $P_{inn}$	$5\times 10^{-5}$	1/cell	Estim.
12	$\gamma_{A_{V}}$	Parameter used in $P_{A_{V}}$, the probability that a new adaptive immune cell is antiviral	2	–	Estim.
13	$k_{I}$	Killing rate of infected cells by innate immune cells	0.02	cell${}^{-1}$h${}^{-1}$	Estim.
14	$k_{VZ}$	Killing rate of virions by innate immune cells	0.005	cell${}^{-1}$h${}^{-1}$	Est., [33]
15	$m_{I}$	Propensity for an innate immune cell to move toward an infected cell	2	–	Estim.
16	$m_{A_{T}}$	Propensity for an antitumor adaptive immune cell to move toward a tumor cell	2	–	Estim.
17	$m_{A_{V}}$	Propensity for an antiviral adaptive immune cell to move toward an infected cell	2	–	Estim.
18	$a_{Z}$	Rate of infected cell-mediated proliferation of innate immune cells	$2.4\times 10^{-6}$	cell${}^{-1}$h${}^{-1}$	Estim.
19	$\delta_{Z}$	Death rate of innate immune cells	0.008	h${}^{-1}$	[6]
20	$\delta_{YT}$	Death rate of tumor-specific adaptive immune cells	$3.75\times 10^{-4}$	h${}^{-1}$	[23,34]
21	$\delta_{YV}$	Death rate of virus-specific adaptive immune cells	$5.54\times 10^{-3}$	h${}^{-1}$	[23]
22	$\kappa_{Z}$	Number of possible infected cell kills for an innate immune cell	10	cells	Estim.
23	$\kappa_{A_{T}}$	Number of possible tumor cell kills for an antitumor T cell	10	cells	Estim.
24	$\kappa_{A_{V}}$	Number of possible infected cell kills for an antiviral T cell	10	cells	Estim.
25	$r_{mov_{Z}}$	Rate at which an innate immune cell moves to an empty neighboring site	0.05	h${}^{-1}$	Estim.
26	$r_{mov_{AT}}$	Rate at which an antitumor adaptive immune cell moves to an empty neighboring site	0.01	h${}^{-1}$	Estim.
27	$r_{mov_{AV}}$	Rate at which an antitumor adaptive immune cell moves to an empty neighboring site	0.01	h${}^{-1}$	Estim.
28	$a_{AZ}$	Activation rate of adaptive immune cells via innate immune cells	0.05	h${}^{-1}$	Estim.
29	$k_{TA}$	Killing rate of tumor cells by tumor-specific adaptive immune cells	$\frac{1}{24}$	cell${}^{-1}$h${}^{-1}$	[23]
30	$k_{IA}$	Killing rate of infected cells by virus-specific adaptive immune cells	$\frac{1}{24}$	cell${}^{-1}$h${}^{-1}$	[23]
31	$k_{VA}$	Killing rate of virions by virus-specific adaptive immune cells	$10^{-5}$	cell${}^{-1}$h${}^{-1}$	Estim.
32	$a_{AT}$	Rate of tumor cell-mediated proliferation of tumor-specific adaptive immune cells	0.0016	cell${}^{-1}$h${}^{-1}$	[23]
33	$a_{AV}$	Rate of infected cell-mediated proliferation of virus-specific adaptive immune cells	0.025	cell${}^{-1}$h${}^{-1}$	[23]
34	$K_{YQ}$	Inhibition of T cells by PD-1/PD-L1	$1.565\times 10^{-22}$	μmol${}^{2}$/cm${}^{6}$	[35],Est.
35	$\rho_{p}$	Molar concentration of PD-1 per T cell	$8.999\times 10^{-15}$	μmol/cm${}^{3}$	[31], Est.
36	$\rho_{L}$	Molar concentration of PD-L1 per T cell	$1.793\times 10^{-14}$	μmol/cm${}^{3}$	[31], Est.
37	$\epsilon_{T}$	Expression of PD-L1 on tumor cells vs. T cells	10	–	Estim.
38	$\epsilon_{Z}$	Expression of PD-L1 on innate immune cells vs. T cells	10	–	Estim.
39	$\delta_{A}$	Decay rate of anti-PD-1	0.0019	h${}^{-1}$	[30,31]
40	$\mu_{PA}$	Anti-PD-1 blocking rate of PD-1	$1.86\times 10^{7}$	${\mathrm{cm}}^{3}/\mu \mathrm{mol}/h$	[35]
